# The clinical outcomes and surgical strategy for cervical spine tuberculosis: A retrospective study in 78 cases

**DOI:** 10.1097/MD.0000000000011401

**Published:** 2018-07-06

**Authors:** Xin Hua Yin, Bao Rong He, Zhong Kai Liu, Ding Jun Hao

**Affiliations:** Department of Spine Surgery, Hong Hui Hospital, Xi’an Jiaotong University College of Medicine, Xi’an, P.R. China.

**Keywords:** anterior, cervical tuberculosis, posterior, surgical strategy

## Abstract

Literature on the treatment of cervical spinal tuberculosis (CSTB) is uncommon, the surgical approaches to cervical spinal tuberculosis were controversial. The aim of the study was to evaluate the clinical outcomes of 3 surgical techniques in CSTB patients, and to determine the most appropriate approach for CSTB patients. Between April 2006 and June 2012, we performed a retrospective review of clinical and radiographic data that were collected from 850 consecutive spinal tubercular patients, including 87 patients who were diagnosed and treated for CSTB in our hospital. Apart from 9 patients being treated conservatively, the remainder (78 cases) underwent surgery by anterior debridement, interbody fusion and instrumentation (A group), posterior instrumentation and anterior debridement, fusion and instrumentation in a single or two-stage operation (AP group), or posterior debridement, fusion and posterior instrumentation (P group). The patients were evaluated preoperatively and postoperatively on the basis of hematologic, radiographic examinations, and neurologic function. The 78 patients were followed up for a mean duration of 41.2 ± 7.2 months (range, 24–65 months). Postoperatively, the preoperative erythrocyte sedimentation rate (ESR) value returned to normal within 3 to 6 months in all patients, and solid bone fusion was achieved in 3 to 8 months. The patients exhibited significant improvement in deformity and neurological deficit postoperatively, while the visual analog scale for pain showed significant improvement in all patients at the last follow up visit. The follow-up outcomes demonstrated that all 3 surgical methods were viable management options for CSTB. Individualized therapeutic strategies should be selected according to the patient's general condition, focal characteristics, and the surgeon's experience.

## 1 Introduction

As HIV infection and drug resistance increases, the incidence of spinal tuberculosis has increased in both developing and developed countries.^[1]^ The cervical spinal tuberculosis is relatively rare, and the incidence of cervical spinal tuberculosis (CSTB) varies from 2% to 12%.^[2–6]^ However, due to the small cross-sectional diameter of the cervical spinal canal, insidious onset of the disease, delay in diagnosis, and drug resistance, CSTB may lead to severe neurological complications, cervical instability, and invasion of the nerve root and vertebral artery. For this reason, cervical tuberculosis should be diagnosed early and treated rapidly. The management of CSTB can constitute a variety of approaches, ranging from anti-tuberculous therapy to surgery. Despite chemotherapy being the cornerstone of TB treatment, patients with spinal tuberculosis that were treated with chemotherapy alone, suffered an increase of 15° malformation evolution on average, and 3% to 5% of such patients eventually ended up with kyphosis that was greater than 60°.^[7]^ Surgery is required in patients with CSTB in cases of spinal deformity, significant neurological dysfunction, and failure of conservative treatment. Literature on CSTB is scarce and the surgical approach for the treatment of CTSB remains controversial. There is no standard management approach reported in the currently available literature. In this paper, we explore the relevant therapeutic strategies in the management of CSTB.

## 2 Patients and methods

This study was approved by the Ethics Committee of the Hong Hui Hospital of Xi’an Jiaotong University College of Medicine. Between April 2006 and June 2012, a consecutive series of 850 patients with the diagnosis of CSTB was treated in our hospital. Among them, 87 cases were radiologically confirmed as CSTB, and 9 of these patients were treated conservatively, while the remainder (78 cases) underwent surgery. The regions involved ranged from C0 to C7. The American Spinal Injury Association (ASIA) classification was used to assess the neurological function.^[8]^ Accordingly, 16 patients belonged to grade B, 38 to grade C, 23 to grade D, and 1 to grade E. The visual analog scale (VAS) was used to assess pain preoperatively and at the last follow-up visit.^[9]^

The diagnosis of cervical tuberculosis was based on clinical presentation, radiologic findings, hematologic examination, a therapeutic response to anti-tuberculosis therapy, and pathological examination. Laboratory procedures, including the tuberculin test, T-SPOT test, a blood complete count, erythrocyte sedimentation rate (ESR), C-reactive protein, and liver and renal function tests were performed. Routine radiologic procedures included chest radiograph, plain radiographs, computed tomography (CT), and magnetic resonance imaging (MRI). The principles of treatment for cervical tuberculosis were as follows: the 9 patients with minimal vertebral body destruction, who had early tuberculosis with or without minimal abscess, were treated conservatively. Indications for surgery were spinal instability, severe and/or progressive kyphosis, neurological deficit, no response to chemotherapy, and large paraspinal abscess.^[10]^ Apart from the 9 patients that were treated conservatively, the remaining patients (n = 78) underwent surgery, using the anterior, posterior, and combined anteroposterior approaches. The choice of surgical procedure depended on several factors, including the nidus characteristics, patient's general condition, and surgical expertise. However, there was no well-defined selection criteria preoperatively.

### 2.1 Preoperative management

The patients were administrated the following antituberculosis drugs: isoniazid (5 mg/kg), rifampicin (15 mg/kg), ethambutol (15–25 mg/kg), and pyrazinamide (15–30 mg/kg) before the surgery. ESR and temperature were confirmed to be normal or significantly decreased prior to commencement of the surgical procedure. Immediate surgery was recommended for patients with a sudden onset of complete paralysis or respiratory obstruction due to a large abscess. Enhancement of nutrition and correction of anemia and hypoproteinemia were routinely carried out. We used preoperative halo traction (1–3 kg) for patients with a relatively severe kyphosis deformity. None of the patients in this study were HIV-positive.

### 2.2 Operative procedure

**Figure 1:**
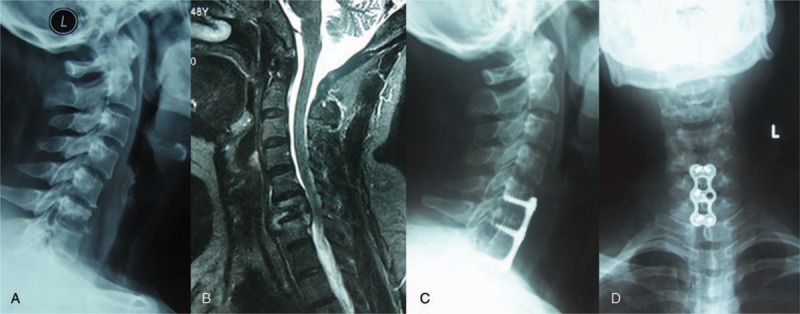
Female, 45 y, C6–7 tuberculosis: x-ray and MRI showed the destruction of vertebral bodies of C6–7 (A and B). The lateral and anteroposterior view of x-ray showed that the anterior infected site had healed and bony union was achieved at the final follow-up (C and D). MRI = magnetic resonance imaging.

The surgery was performed under general anesthesia. If the involvement was confined to a single segment without mass paravertebral abscess, with or without mild kyphosis, then anterior debridement, decompression, bone grafting, and instrumentation were performed. Mild kyphosis could be corrected during anterior instrumentation (Fig. 1).^[11]^

**Figure 2:**
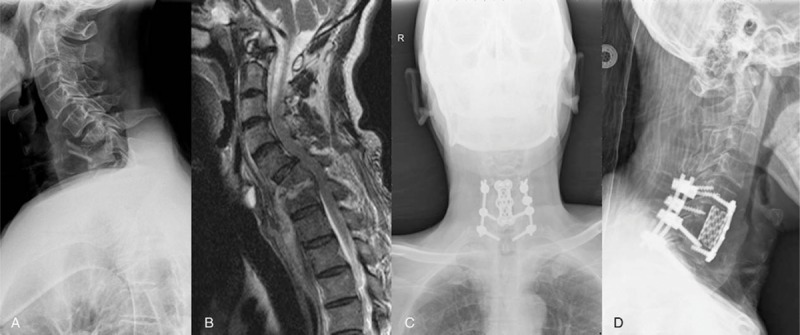
A 48-year-old man was diagnosed as having cervical tuberculous spondylitis (C6–7). X-ray, MRI showed the destruction of vertebral bodies of C6–7 (A–B). Final follow-up radiographs showed good bone fusion (C–D). MRI = magnetic resonance imaging.

In patients with severe kyphosis, with involvement of >2 adjacent segments or with a large paravertebral abscess, anterior focal debridement, bone grafting, instrumentation, and posterior instrumentation was performed in a single stage or in 2 stages, depending on the patient condition (Fig. 2).^[12]^

**Figure 3:**
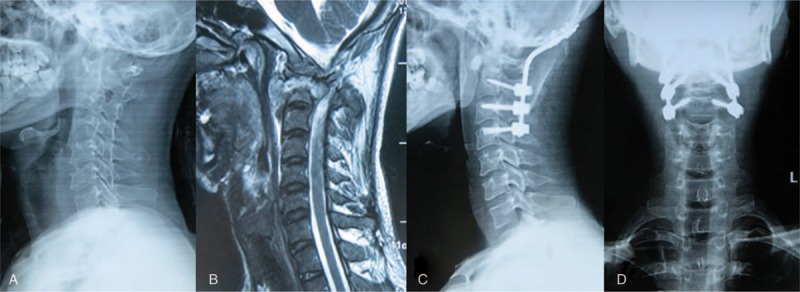
A 27-year-old man was diagnosed as having upper cervical tuberculous spondylitis after a 3 months history of severe cervical pain. X-ray and MRI showed the destruction of vertebral bodies of C0–C2 (A–B), final follow-up radiographs showed good bone fusion (C–D). MRI = magnetic resonance imaging.

Additionally, an upper cervical lesion, with or without mass abscess and vertebral body destruction, could be managed with posterior debridement, decompression, bone grafting, and instrumentation (Fig. 3).^[13]^

### 2.3 Postoperative management

Patients were ambulatory for 2 to 3 days postoperatively, then a brace was provided to each patient, which was continued for at least 3 months until graft union was achieved. The antituberculosis therapy was administered for 12 to 18 months, postoperatively. If the drug sensitivity test results indicated resistance to any first- line drug, chemotherapy was tailored to these patients based on their pervious chemotherapy history and drug-susceptibility profiles. X-ray, ESR, and liver function were evaluated at 1, 2, 3, 6, and 12 months after surgery, and then once a year thereafter. Using SPSS 20.0 software (SPSS, Inc., Chicago, IL), a paired sample *t* test was used to compare the pre- and postoperative clinical and radiographic data, with a *P* value of .05 considered to be statistically significant. The results are reported as mean ± standard deviation (SD).

## 3 Results

The cohort comprised of 43 men and 35 women, with an average age of 36.9 ± 1.6 years (range, 20–79). There were 10 patients who had involvement of vertebrae at multiple levels, and 6 patients who had 2 vertebrae involved. There were 87.5% (68/78) patients who had loss of disc height, and 10 patients showed no obvious change. The vertebral body destruction (predominantly anterior) occurred in 85.2% of the patients (66/78). Well-defined abnormal vertebral signal could be observed in the remaining 9.5%. Paraspinal abscess formation was found in 90% (70/78) of cervical TB patients, and 36 patients had calcification in the paraspinal masses. The average kyphotic angle in the 3 groups was 14.5° ± 7.5°, 30.8° ± 10.5°, and 2.6° ± 12.5°, respectively. Patients were admitted with complaints of neck pain (100%), stiffness/restricted neck range of motion (100%), dysphagia/dyspnea (25%), low-grade fever (45%), progressive torticollis (32%), weight loss (70%), incomplete paraplegia caused by spinal cord injury (99%), discomfort in the throat, and respiratory difficulty (5%) (Table 1). The mean duration of disease was 4.5 ± 1.1 months. The minimum follow-up period was 2 years. A total of 78 patients were treated surgically. For each group, the mean operative time, blood loss, and bone fusion are shown in Table 3.

**Table 1:**
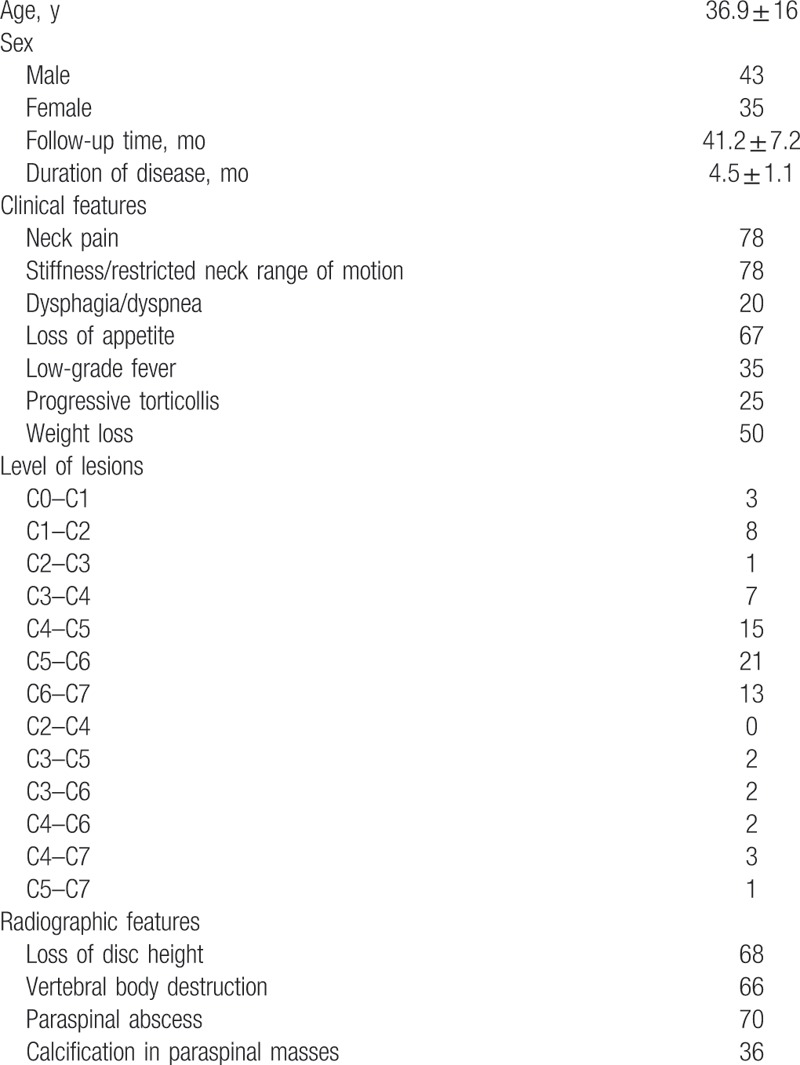
Patient demographic and clinical characteristics.

### 3.1 Neurological status

Postoperatively, there was no increase in neurological deficit. There were 77 cases who suffered neurologic deficit before surgery, and at the last follow-up visit the neurologic deficit in all patients had improved. Five patients showed impaired neurological function postoperatively, which was attributed to delay in diagnosis of CSTB (Table 2).

**Table 2:**
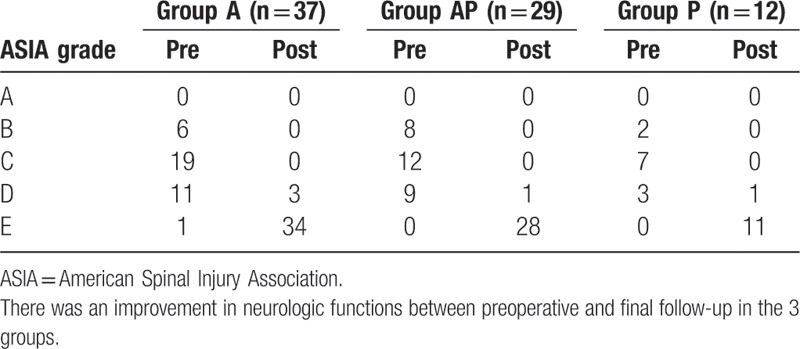
Recovery of neurologic functions.

### 3.2 Clinical outcomes

Neck pain and stiffness had improved in all patients. And the mean VAS score had improved at the last visit (Table 3).

**Table 3:**
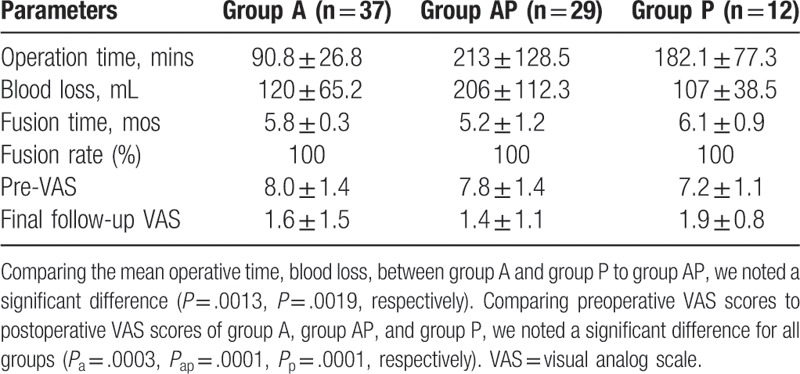
Clinical details of surgery.

### 3.3 Kyphosis deformity and erythrocyte sedimentation rate results

In this series, the ESR returned to normal in all patients, within 3 to 6 months after surgery. The average degree of kyphosis was 14.5° ± 7.5° in group A, 30.8° ± 10.5° in group AP, and 2.6° ± 12.5° in group P. They significantly decreased to –4.8° ± 2.1° in group A, −5.1° ± 4.0° in group B, and −13.2 ± 10.6° in group C postoperatively They had significantly improved in comparison to the preoperative measurements (*P*_a_ = .003, *P*_ap_ = .002, *P*_p_ = .001). The mean kyphosis angle was –4.6 ± 1.8° in group A, –4.9° ± 3.8° in group AP, and –13.9° ± 17.0° in group P, at the last follow-up. They had no significantly loss of corrected angle in comparison to the postoperative measurements (*P*_a_ = .071, *P*_ap_ = .341, *P*_p_ = .257) (Table 4)

**Table 4:**
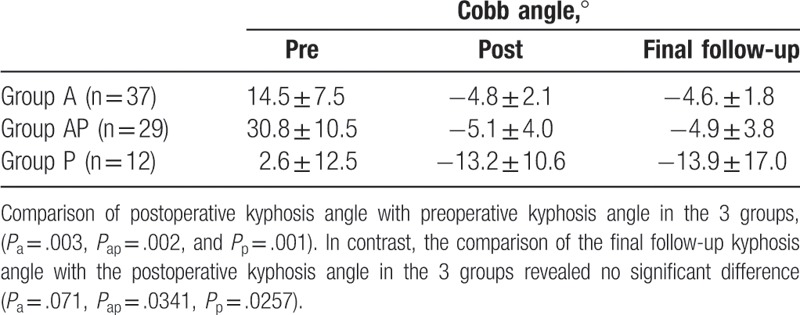
Kyphosis.

### 3.4 Complications

One patient in group B presented with delayed wound healing due to pre-existing diabetes, which improved upon achieving control of the blood sugar levels. In addition, 2 patients suffered from cerebrospinal fluid leakage in group A and group AP. Two patients in group A presented with internal fixation loosening, postoperatively. We had to remove the instrument after fusion. Seven patients reported harvest-site pain postoperatively, but this was managed with physical therapy and acupuncture, and the symptoms had improved at final follow-up. There were 3 patients with a history of gout, who presented with pain and swelling of the joints due to urate accumulation, secondary to pyrazinamide use. The symptoms of gout improved upon cessation of pyrazinamide (Table 5).

**Table 5:**
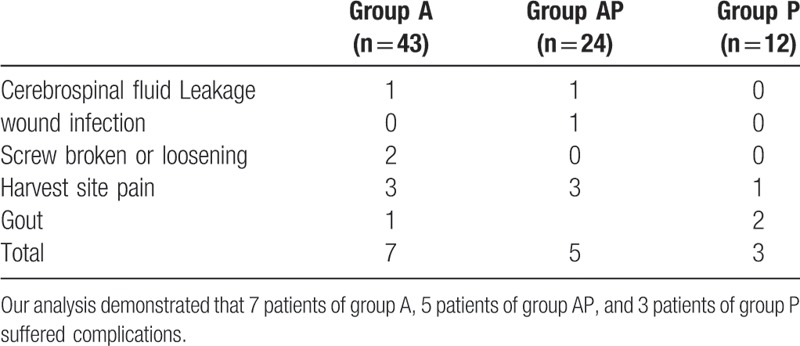
Complications.

## 4 Discussion

In the cervical spine, the line of weight transmission passes through the posterior half of the vertebral bodies. Therefore, CSTB first causes obliteration of natural cervical lordosis followed by later appearance of kyphosis. Because of the high level of mobility and load forces in the cervical spine, the development of symptoms is rapid. In addition, pus, destructed vertebrae, and granulation tissues can induce spinal deformity and spinal cord compression, which can lead to swallowing or breathing difficulties.^[14–16]^

There is a paucity of literature on the treatment of patients with cervical spine tuberculosis. Tuberculosis of the cervical spine is rare, the incidence of CSTB is reported as 4.2% to 12%,^[2–6]^ and it was 9.18% in our study. The mean interval between onset of symptoms and clinical diagnosis was typically around 12 months but has now decreased to between 3 and 6 months.^[17]^ In the present study this interval was 4.5 ± 1.1 months.

Delay in diagnosis is common owing to the fact that the clinical presentation of cervical tuberculosis is indistinct and nonspecific.^[18–20]^ The main symptoms of cervical tuberculosis are neck pain and restricted neck movement.^[21,22]^ Cervical pain was the most common symptom in our study, and was associated with cervical stiffness, both of which showed statistically significant improvement at the last follow-up.

Cervical spine tuberculosis is considered a catastrophic disease due to the associated probability of spinal cord compression and quadriplegia. This emphasizes the importance of early diagnosis in the management of cervical tuberculosis. Although plain x-rays may appear normal in the early stage of disease, they continue to be the initial screening procedure when infectious spondylitis is suspected.^[23,24]^ This is because plain x-ray changes are often present by the time the patient is investigated for the disease.^[25–27]^ Furthermore, preliminary exclusion of other diseases is done based on the x-ray findings. We recommend that x-ray examination be included for preliminary diagnosis of tuberculosis. Previous studies have shown that the most common x-ray findings consist of vertebral body destruction and disc narrowing. However, the height of the disc space may be preserved until the later stages of the infection.^[23]^ Prompt medical and surgical treatment may avert serious complications in CSTB patients. MRI is considered the investigation of choice in spinal infection because it has high sensitivity and satisfactory specificity. The advantages of CT over MRI are that CT offer more reliable detection of calcified foci and they also provide guidance on the need for interventional procedures.

There is little literature at present describing comprehensive therapeutic strategies for cervical spine tuberculosis. The treatment approaches to cervical spine tuberculosis have fluctuated between conservative therapy and surgery.^[28]^ In this paper, we sought to evaluate the outcomes of surgical management (anterior surgery, posterior surgery, and a combination of posterior and anterior surgery) in cervical spine tuberculosis, and to discuss the most appropriate therapeutic strategies.

## 5 Anterior approach

CSTB lesions are located predominantly on the anterior column. Performing a 1-stage anterior debridement and bone grafting fusion with instrumentation, has the advantages of direct access to the focus of the disease, bony union, and stabilization of the spine. Hassan^[29]^ reported the outcomes of 16 patients with lower cervical tuberculosis and neurological complications or unacceptable kyphosis, who underwent 1-stage anterior debridement, bone grafting fusion, and H-plate fixation. At the end of the follow-up period (38 months), all cases showed bony fusion and there were no increased neurological deficits. In 2014, He et al^[11]^ reported the outcomes of 25 patients (18 men and 7 women; average age, 39 years) with lower cervical spine tuberculosis who were treated with anterior debridement, decompression, bone grafting, and instrumentation. Average fusion period was 6.8 months (range, 3–10 months). None of the patients had radiologic findings of internal fixation failure. No patients showed recurrence of tuberculosis infection or draining sinus. In this series, all cases (in group A) achieved satisfactory clinical outcomes, except for 2 patients who presented with loosening of screws. In our experience, if the number of destroyed vertebrae is ≤2, and the patients do not have severe kyphosis, the mono-stage anterior approach can achieve satisfactory outcomes, with shorter operative time and less blood loss. This may be the most appropriate surgical approach to cervical tuberculosis, especially for patients in the early phase of bone destruction and/or with mild/moderate kyphosis.

## 6 Anterior+posterior(AP) approach

It is known that if anterior instrumentation was done over 3 or more residual vertebrae, both proximal and distal screws will have to withstand significantly greater stress, and thus, the screws are more prone to breakage or loosening. In this scenario, we recommend patients undergo a combined anterior and posterior surgery. In 2015, Zeng et al^[12]^ reported the outcomes of 25 cases with cervical tuberculosis that underwent circumferential instrumented fusion (anterior titanium plate and posterior pedicle, or lateral mass fixation) combined with anterior debridement, decompression, and bone fusion. The average follow-up period was 34.1 ± 7.0 months (24–48 months). The authors reported that combined anterior and posterior surgery may provide better correction of the deformity, with more rapid recovery of spinal cord function and less complications. Deng et al^[30]^ suggested that 1-stage combined posterior and anterior approaches should be used for the treatment of severe, active sub-axial cervical tuberculosis, complicated with kyphosis, in children. In their study, between 2008 and 2013, 13 children (with a mean age of 8.7 years) suffered from active sub-axial cervical tuberculosis, which was complicated by kyphosis, all of whom underwent a 1-stage combined posterior and anterior approach. The mean Cobb angle of cervical kyphosis was 30.8 ± 10.5° in the preoperative stage, −5.1 ± 4.0° in the postoperative stage, and –4.9 ± 3.8° at final follow-up. In our study, 29 patients underwent a combined anterior and posterior procedure, and the follow-up results revealed favorable outcomes. In our experience, anterior debridement and fusion is considered to be a better approach in view of decompression and fusion. In cases with multilevel involvement, an anterior debridement is mandatory. If >2 consecutive vertebrae are severely damaged, especially in patients with severe kyphosis or in the pediatric age group, a combined anterior and posterior approach is mandatory. In such cases, posterior instrumentation plays an important role in the partial correction of kyphosis and in preventing the breakage or loosening of the screws. When the Cobb angle of cervical kyphosis is >30°, the patients should receive a maintained halo traction of 3 kg, in order to decrease the kyphosis deformity. These approaches not only provide vertebral reposition (restoration of physiological curvature and intervertebral height of cervical spine), but also lead to complete spinal decompression. However, the AP approach is not devoid of disadvantages. The results of the present study demonstrate that the use of 2 positions and 2 incisions lead to a longer operating time (213 ± 128.5 minutes), increased amount of bleeding (206 ± 112.3 mL), and larger wounds. In addition, the AP approach may not be tolerated well by patients with a poor general state of health.

## 7 Posterior approach

There are some reports about the use of the posterior surgical approach in cases of cervical spine tuberculosis.^[31,32]^ Zhang et al^[33]^ reported positive results after a 1-stage posterior surgery in 11 patients with upper cervical spine tuberculosis. In the study, the average follow-up period was 28.1 ± 10.5 months. There were no postoperative complications, related to instrumentation, and neurologic function had improved at the last follow-up. All patients achieved bony fusion within 3 to 8 months following surgery. In 2014, Wang et al also reported favorable outcomes in patients with CSTB who underwent posterior radical debridement, decompression, and internal fixation. Mandavia et al^[34]^ demonstrated that a posterior-only cervical approach in patients with atlantoaxial tuberculosis resulted in successful outcomes. In our study, 12 patients underwent posterior debridement, fusion, and fixation surgery. The surgical wounds healed without chronic infection or sinus formation. The mean Cobb angle of kyphosis was 2.6 ± 12.5° in the postoperative stage, and −13.9 ± 17.0° at the last follow-up visit. All patients showed significant improvement following surgery. In our opinion, a posterior-only surgical approach is the appropriate in the following cases: patients with upper cervical spinal tuberculosis with no extensive abscess; when chemotherapy fails and there is no obvious vertebral collapse or spinal cord compression; cervical vertebral accessories tuberculosis.

The management of CSTB is challenging and controversial. The most appropriate surgical strategy varies according to the characteristics of each patient (age, extent of vertebrae destruction, differences in patient demands for resolution of their complaints, extent of abscess, and patient's general condition). Differences in defining the criteria for varying management strategies, among practitioners, limit the usefulness of our study.

## 8 Conclusion

The results of this study indicate that posterior, anterior–posterior, and anterior surgical approaches are all satisfactory options for the management of cervical tuberculosis and have their own relative indications. Individualized surgical strategies should be formulated according to the different characteristics of CSTB patients.

## Author contributions

**Data curation:** Baorong He.

**Investigation:** Ding jun Hao.

**Writing – original draft:** Xinhua Yin Yin.

**Writing – review & editing:** Zhongkai Liu.
